# The role of small extracellular vesicles and microRNA as their cargo in the spinal cord injury pathophysiology and therapy

**DOI:** 10.3389/fnins.2024.1400413

**Published:** 2024-05-07

**Authors:** Kristyna Sintakova, Nataliya Romanyuk

**Affiliations:** ^1^Department of Neuroregeneration, Institute of Experimental Medicine of the Czech Academy of Sciences, Prague, Czechia; ^2^Department of Neuroscience, 2nd Faculty of Medicine, Charles University, Prague, Czechia

**Keywords:** spinal cord injury, regeneration, stem cells, small extracellular vesicles, miRNA

## Abstract

Spinal cord injury (SCI) is a devastating condition with a complex pathology that affects a significant portion of the population and causes long-term consequences. After primary injury, an inflammatory cascade of secondary injury occurs, followed by neuronal cell death and glial scar formation. Together with the limited regenerative capacity of the central nervous system, these are the main reasons for the poor prognosis after SCI. Despite recent advances, there is still no effective treatment. Promising therapeutic approaches include stem cells transplantation, which has demonstrated neuroprotective and immunomodulatory effects in SCI. This positive effect is thought to be mediated by small extracellular vesicles (sEVs); membrane-bound nanovesicles involved in intercellular communication through transport of functional proteins and RNA molecules. In this review, we summarize the current knowledge about sEVs and microRNA as their cargo as one of the most promising therapeutic approaches for the treatment of SCI. We provide a comprehensive overview of their role in SCI pathophysiology, neuroprotective potential and therapeutic effect.

## 1 Introduction

Spinal cord injury (SCI) is a life-changing condition that leads to devastating neurological impairment and affects a significant portion of the population each year. Consequences of SCI include temporary and permanent changes in spinal cord function, as well as loss of motor, autonomic, and sensory functions (Dumont et al., 2001; Alizadeh et al., 2019). Global prevalence of SCI is approximately 20.6 million cases, with 250.000–500.00 new cases every year. The cause of SCI can be of non-traumatic, e.g., due to vascular ischemia or tumor; or, more commonly, SCI can be of traumatic origin, caused by an external force (McKinley et al., 1999; Ahuja et al., 2017a). Causes include traffic accidents, violence and sport related injuries in younger individuals. In older individuals, with underlying age-related degenerative changes, the main cause of SCI is low-energy impact, usually from a fall. SCI happens more frequently in adults than in children. Prevalence of SCI is roughly equal in males and females under 5 years of age, but more common in males than in females in older age groups; with men being affected during early adulthood and women during adolescence, and with both affected during late adulthood (Vogel and Anderson, 2003; Hachem et al., 2017; Ahuja et al., 2017b; Alizadeh et al., 2019; Ding et al., 2022). Neurological recovery potential and rate of recovery in pediatric patients with traumatic SCI is considered better when compared to adults. Pediatric spinal cord is more elastic and its response to SCI shows different injury patterns when compared to adult SCI (Ding et al., 2022; Cunha et al., 2023). In terms of prognosis, whether the spinal cord is severed completely or incompletely is important (Oyinbo, 2011). In most cases, the spinal cord is not severed completely (Tator and Fehlings, 1991).

Despite tremendous advances in diagnosis and survival rates in recent years, an effective treatment for SCI is still not available. The poor prognosis after SCI is due to the inhibitory properties of the glial scar, poor remyelination, and limited regenerative capacity of the central nervous system (CNS). The adult spinal cord has only a very limited ability to form new neurons or regenerate damaged nerve cells. In recent years, it has been shown that the CNS has a greater regenerative capacity than previously thought, but its capacity is still very limited and weaker than that of the peripheral nervous system (PNS), and also decreases with age (Ahuja et al., 2017a; Hayta and Elden, 2018).

**Figure 1:**
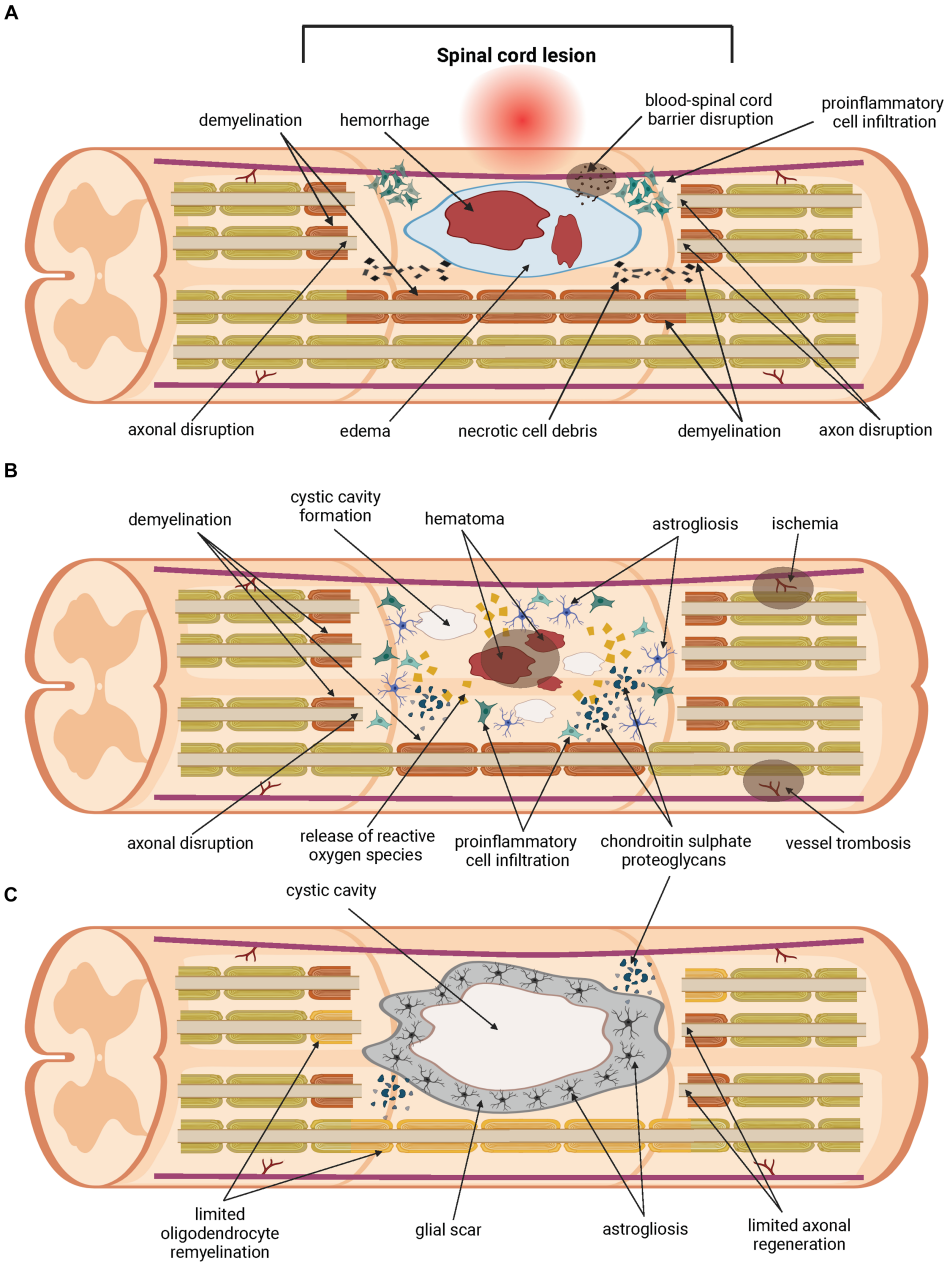
Pathology of the spinal cord injury. Traumatic primary injury progresses into inflammatory cascade of secondary injury within hours, lasts for weeks or longer, and can be divided into three phases. The acute phase of the secondary spinal cord injury **(A)** develops within the first few days post-injury. Disruption of the blood-spinal cord barrier (BSCB) occurs, followed by hemorrhage, edema and demyelination. Homeostatic balance is disturbed. Pro-inflammatory cells migrate from the periphery to the site of injury. The release of pro-apoptotic factors such as cytokines, ions, and parts of apoptotic cells contributes to ongoing cell death. Damage to neurons and oligodendrocytes leads to further loss of their function. Chondroitin sulphate proteoglycans (CSPGs) are released by activated astrocytes. The acute phase transitions to sub-acute **(B)**, which lasts up to two weeks post-injury. During this phase, further apoptotic and necrotic cell death of neurons and oligodendrocytes occurs. The inflammatory environment is exacerbated by the ongoing pathological processes, and damage spreads into healthy tissue surrounding the lesions. Release of cytokines, nucleic acids (DNA), adenosine triphosphates (ATP) and reactive oxygen species (ROS), ions, and parts of apoptotic cells contributes to ongoing cell death, demyelination and axon disruption. Injury transitions into chronic phase **(C)**. This phase lasts from weeks up to six months. Cystic cavities merge and impede regeneration. A glial scar is formed, containing a large number of CSPGs, which acts as a physical and biochemical barrier, preventing the growth of neurites and limiting the passage of cells mediating regeneration. Oligodendrocyte remyelination and axonal regeneration are limited severely.

Another challenge in the SCI treatment is the complexity of the injury itself (Figure 1). A traumatic primary injury is followed by an inflammatory cascade of secondary injury that leads to a progressive damage to spinal cord tissue, developing after the primary injury within minutes and lasting for several weeks, or even months. Secondary injury can be divided into acute (approximately 0–48 h up to 4 days post-injury), subacute (from approximately 2–4 days up to two weeks post-injury) and chronic (from two weeks post-injury, lasting up to six months post-injury) (Ahuja et al., 2017b; Alizadeh et al., 2019). Ongoing pathological processes include ischemia, neuroinflammation, disturbance of ionic homeostasis, demyelination, apoptotic and necrotic cell death. Inflammatory cells migrate from the periphery to the site of injury, followed by the release of pro-inflammatory cytokines and cytotoxic particles such as nucleic acids (DNA), adenosine triphosphates (ATP) and reactive oxygen species (ROS). Disruption of the blood-spinal cord barrier (BSCB) during the primary injury leads to the accumulation of inflammatory cytokines. All of these processes contribute to an inflammatory environment at the site of injury and to the spread of damage to initially healthy tissues surrounding the lesion, thereby increasing the neurological deficit. As the secondary injury progresses, the acute phase becomes sub-acute and then chronic. Axons are demyelinated, neurons and oligodendrocytes die by apoptotic cell death and further cell necrosis occurs. Regeneration is impeded by cystic cavitation and glial scar formation (Hausmann, 2003; Oyinbo, 2011; Amo-Aparicio et al., 2018; Hayta and Elden, 2018; Ren et al., 2018; Tran et al., 2018). Physiological function of the glial scar is to serve as a protective barrier that forms around the site of the injury and prevents the spread of inflammation into the surrounding healthy tissue. Its heterogenous structure is formed by many cell types within the injured spinal cord, mainly by activated astrocytes secreting chondroitin sulphate proteoglycans (CSPGs) (McKeon et al., 1991; Pekny and Nilsson, 2005). Proliferation of activated astrocytes stops in approximately 1–2 weeks, signaling maturation of the glial scar (Clifford et al., 2023). Regardless of the protective role of the glial scar in acute SCI, its presence significantly hinders regeneration, axonal growth and effective treatment in later, chronic phases of SCI. Thus, the glial scar appears to be an ideal target for therapeutic intervention in the treatment of SCI (Yuan and He, 2013; Cregg et al., 2014). However, preventing the glial scar formation does not promote axonal regeneration, nor it leads to better outcome in SCI (Anderson et al., 2016).

Current therapeutic approaches to SCI are aimed at mitigating the symptoms and reducing the consequences of SCI, such as early surgical decompression (Fehlings et al., 2012), blood pressure augmentation (Wilson et al., 2013), therapeutic hypothermia (Kwon et al., 2008), or cerebrospinal fluid (CSF) drainage to prevent hypoperfusion of the spinal cord in the critical period after the injury by reducing the pressure (Martirosyan et al., 2015). To date, administration of methylprednisolone sodium succinate, a synthetic glucocorticosteroid, is the only approved drug for SCI treatment. In combination with surgical decompression, it is a primary pharmacological SCI treatment in clinical use (Fehlings et al., 2017). Other experimental compounds with neuroprotective and immunomodulatory properties in pre-clinical studies include corticosteroid bacteriostatic antibiotic minocycline (Festoff et al., 2006; Donnelly and Popovich, 2008), riluzole (Azbill et al., 2000; Simard et al., 2012), curcumin (Ni et al., 2015; Lin et al., 2017), epigallocatechin gallate (EGCG) (Machova Urdzikova et al., 2017), and fibroblast growth factor (FGF) (Zhou et al., 2018). Tissue engineering is also being explored as a potential therapeutic approach (Dumont et al., 2016), as well as optogenetics (Ahmad et al., 2015).

Another potential therapeutic strategy in the SCI treatment is administration of rapamycin, an inhibitor of mTOR kinase with neuroprotective properties (Li X. G. et al., 2019). mTOR is an evolutionary conserved serine/threonine protein kinase of intracellular PI3K/Akt/mTOR signaling pathway, integrating environmental cues that are important for growth regulation and homeostasis. Abnormalities in mTOR pathway regulation can lead to pathologies. The opposite is also true – the pathway is activated in pathologies such as spinal cord injury (Saxton and Sabatini, 2017). These properties make the mTOR a suitable target for therapeutic intervention using immunosuppressants, such as rapamycin, which has demonstrated neuroprotective effect in neurodegenerative diseases, including SCI. Inhibition of mTOR signaling pathway after SCI resulted in increased autophagy and significant reduction in neuronal loss and cell death in the injured spinal cord (Erlich et al., 2007; Kanno et al., 2012; Zhang et al., 2014; Li X. G. et al., 2019).

Modulation of autophagy is another experimental therapeutic approach in the SCI treatment. Autophagy itself is an important physiological mechanism with neuroprotective function, and its disruption during SCI contributes to the development of secondary injury and neuronal loss (Liu et al., 2015). Due to the accumulation of autophagosomes after SCI, autophagic markers are increased after SCI. However, the reason for this is not autophagy itself, but the inhibition of autophagy flux. This leads to impaired lysosome function and the autophagosome degradation process after SCI, which may lead to greater cellular stress and neuronal cell death (Liu et al., 2015; Zhou et al., 2017). Studies have demonstrated that modulation of autophagy has neuroprotective effects in SCI, supports functional recovery and inhibits neuronal apoptosis (Wang et al., 2014; Zhang et al., 2014; Li et al., 2021).

Cell transplantation is one of the most promising approaches to SCI treatment. Various cell types demonstrated immunomodulatory and neuroprotective properties, for example Schwann cells (Wiliams and Bunge, 2012) or various types of stem cells. Mesenchymal stem/stromal cells (MSCs) modulate inflammation at both local and systemic level in SCI by inhibiting the activity of pro-inflammatory cells and factors, thus reducing inflammatory response in the lesion area following SCI. Studies have shown that MSC transplantation induced modulation of macrophage phenotype from pro-inflammatory M1 to protective M2, supported motor function recovery and glial scar reduction (Nakajima et al., 2012; Quertainmont et al., 2012). MSCs can be used in combination with biomaterials, which provide structural support or serve as carriers to ensure stem cell delivery (Cofano et al., 2019). Li et al. described how bone marrow-derived stem/stromal cells (BMSCs) extended their protective effect on post-injury tissue through mitochondrial transfer via gap junctions into injured neurons, using oxygen–glucose deprivation injured neurons as a model, as well as animal SCI rat model. The internalization of mitochondria led to an improvement in the bioenergetic profile, decreased apoptosis and promoted cell survival in motor neurons post oxygen–glucose deprivation. When transplanted into injured spinal cord of SCI rats, both mitochondrial and BMSC transplantation resulted in improved locomotor functional recovery (Li H. et al., 2019).

In comparison to MSC transplantation, neural stem cells (NSCs) and neural progenitor cells (NPCs) have yielded better results in SCI pathology, with locomotor function recovered and glial scaring reduced (Ruzicka et al., 2017). Therapeutic potential of NSCs a NPCs has also been investigated with promising results in other CNS pathologies, including neurodegenerative diseases (Bonnamain et al., 2012), stroke (Nakagomi et al., 2019), traumatic brain injury (TBI) (Ludwig et al., 2018). NSCs are multipotent stem cells that can give rise to cell types of the neural lineage, such as neurons, oligodendrocytes and astrocytes (Reynolds and Weiss, 1992; Andreotti et al., 2019). Differentiation into neurons occurs mainly during embryonic development, gliogenesis predominates postnatally. In adulthood, neurogenesis prevails in the subventricular zone (SVZ) and subgranular zone (SGZ) of the hippocampus (Ihrie and Alvarez-Buylla, 2008; Beattie and Hippenmeyer, 2017). NSCs can also be derived from pluripotent stem cells such as embryonic stem cells (ESCs) or induced pluripotent stem cells (iPSCs). Due to their neuroprotective and immunomodulatory properties and therapeutic plasticity, transplantation of NSCs or NPCs is considered as a very promising approach to the treatment of various CNS pathologies and diseases. Several studies have shown that NSC transplantation supports regeneration, synaptic plasticity and functional recovery after SCI, promotes axonal sprouting and remyelination (Parr et al., 2007; Martino et al., 2011; Romanyuk et al., 2015; Salewski et al., 2015; Pereira et al., 2019), as well as modulation of inflammatory response through reducing activation of M1 macrophages, and decreases mRNA levels of pro-inflammatory cytokines (Cheng et al., 2016).

There are two approaches to NSCs and NPCs treatment in SCI: either exogenous transplantation, or activation of endogenous NSCs, which are present in the adult spinal cord and can be mobilized to support regenerative mechanisms after SCI in rat spinal cord (Moreno-Manzano et al., 2009). Exogenous transplantation of various NSC types exhibited immunosuppressive and anti-inflammatory properties through inhibition of NF-κB pathway, which is involved in the inflammatory response after SCI; further reduced astrogliosis, and had a beneficial effect on recovery, axonal sprouting and preservation of grey and white matter after SCI (Amemori et al., 2015; Karova et al., 2019). Both approaches can be combined with biomaterials, scaffolds of nanoparticles (Cheng et al., 2021; Zou et al., 2022). A persistent problem with NSC transplantation is a low survival rate due to ischemia (Balsam et al., 2004). Other limitations of direct NSC transplantation include cell dedifferentiation, immune reaction and transplant rejection, and tumorigenesis (Jeong et al., 2011).

The positive effect of stem cell transplantation is undeniable; however, its exact mechanism has not yet been fully elucidated. Recent studies have shown that transplanted stem cells contribute to the functional recovery of the spinal cord not directly by proliferation and differentiation into cell types of neural cell line, but rather by their paracrine effect, which seems to be mediated by small extracellular vesicles (sEVs) secreted from transplanted stem cells (Baglio et al., 2012; Maumus et al., 2013; Stevanato et al., 2016; Chang et al., 2018; Rong et al., 2019b; Forostyak et al., 2020; Zhong et al., 2020); and, more precisely, through small non-coding RNA molecules (ncRNAs) encapsulated within these vesicles (Wiklander et al., 2019).

## 2 Small extracellular vesicles and miRNA

**Figure 2:**
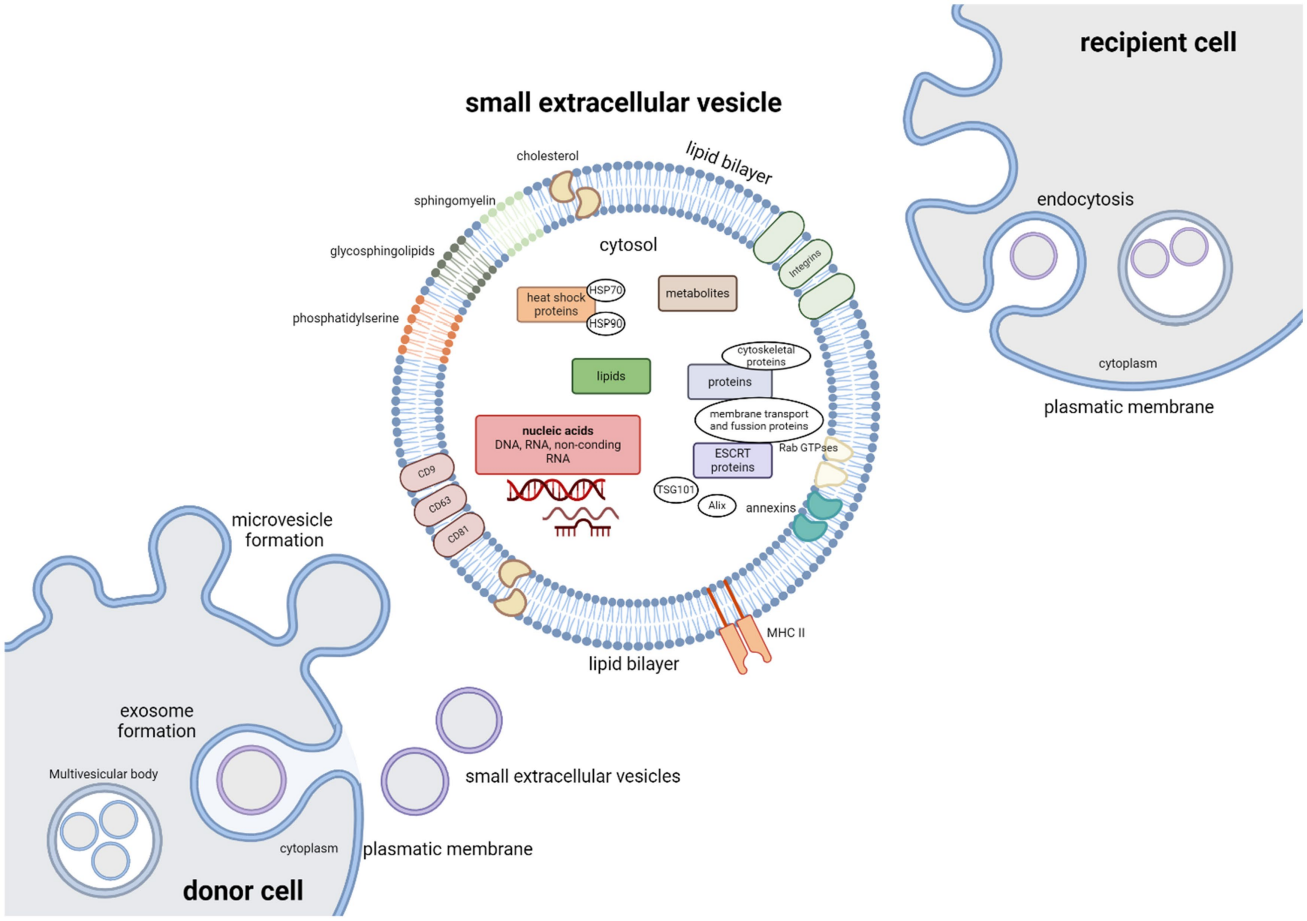
Small extracellular vesicles – cargo, biogenesis and uptake. The cargo of extracellular vesicles consists of many different biomolecules: lipids, metabolites, nucleic acids (DNA, RNA), and functional proteins including tetraspanins (CD9, CD63, and CD81), cytoskeletal proteins, annexins, proteins participating in membrane transport and fusion (Rab GTPases), heat shock proteins (HSP70 and HSP90), endosomal sorting complex required for transport (ESCRT) proteins that are crucial for vesicle biogenesis, ESCRT complex-related proteins (Tsg101, Alix), major histocompatibility complex (MHC) II and integrins. Vesicle membranes are rich in glycosphingolipids, cholesterol, sphingomyelin and phosphatidylserine. Microvesicles are formed by outward-budding of the plasma membrane and simply released. Exosome biogenesis can be divided into three phases. Invagination of the plasma membrane is followed by formation of multivesicular body (MVBs) by inward budding of the endosomal membrane, followed by exosome release as MVBs fuse with the plasma membrane. Vesicles can be taken up by the recipient cells by either interaction between surface receptors and ligands of vesicles and recipient cell, by direct fusion between vesicle and recipient cell plasma membranes, or by endocytosis.

Small extracellular vesicles are nanometer-sized (approximately 50–200 nm in diameter), endocytic membrane bound nanovesicles. sEVs are a very heterogenous group, with vesicle subtypes varying in size, biogenesis pathway, cell of origin, cargo and function. Surface proteins and cargoes vary, as do the relative amounts produced by different cells, but they all produce them. These vesicles subtypes include exosomes, microvesicles, oncosomes, apoptotic bodies, and others (Théry et al., 2018; Bazzan et al., 2021). Originally thought to be just an instrument of selective cellular waste disposal (Johnstone et al., 1991), they can be found in all body fluids and are released from almost all cell types under both normal and pathological conditions. sEVs play a crucial role as mediators of intercellular communication between different cell types by transporting functional proteins, lipids, metabolites and nucleic acids (DNA, RNA and other ncRNA molecules) to recipient cells. The composition of sEVs cargo is very complex and consists of hundreds to thousands of different biomolecules (Raposo and Stoorvogel, 2013; Colombo et al., 2014; Gurunathan et al., 2019) that are involved in many cellular and pathological processes (Figure 2). These include blood coagulation, both innate and adaptive immune response, angiogenesis, various aspects of cell communication within the nervous system, cell development and death, inflammation, tumorigenesis and neurodegenerative diseases (Maas et al., 2017; Van Niel et al., 2018; Gurunathan et al., 2019; Dutta et al., 2021). sEVs protein components consist of tetraspanins (CD9, CD63 and CD81), cytoskeletal proteins, annexins, proteins participating in membrane transport and fusion (Ras-associated binding (Rab) GTPases), heat shock proteins (HSP70 and HSP90), endosomal sorting complex required for transport (ESCRT) proteins that are crucial for vesicle biogenesis, ESCRT complex-related proteins (Tsg101, Alix), and integrins. Protein content can be influenced by the producer cell, for example antigen presenting cells-derived sEVs can carry major histocompatibility complex (MHC) II; as well as by the cell microenvironment (Colombo et al., 2014; Van Niel et al., 2018; Jeppesen et al., 2019; Lim et al., 2023). The cargo to be encapsulated in vesicles is selected by sorting mechanisms; however, not all proteins involved in this process nor their mechanism of effect have yet been fully elucidated (Noltet Hoen et al., 2012).

Extracellular vesicles are formed either by an outward-budding of the plasma membrane – this type of vesicles is referred to as microvesicles – or by lipid curvature forming an inward-budding vesicle within the endocytic system, in which case the emerging vesicles are called exosomes. Microvesicles and exosomes differ in both biogenesis pathway and size. Exosome biogenesis can be divided into three phases. Formation of vesicles by invagination of the plasma membrane is followed by the formation of multivesicular bodies (MVBs) by inward budding of the endosomal membrane (Figure 2). Mechanism of exosome biogenesis includes recruitment of ESCRT machinery, which consists of four protein complexes – ESCRT-0, -I, -II, and-III. These protein complexes, along with accessory proteins such as Alix, vacuolar protein sorting-associated protein 4 (VSP4), and vesicle trafficking 1 (VTA1) form intraluminal vesicles (ILVs) and incorporate cargo into emerging exosomes. Exosomes are then released as MVBs fuse with the plasma membrane, while microvesicles are released from the parent cell by simply pinching off the plasmatic membrane. Alternatively, MVBs can fuse with lysosomes with help from autophagosomes. This leads to degradation and recycling of their cargo and possible autocrine signaling. Molecules involved in release of exosomes include proteins of the Rab family (Rab7, Rab11, Rab27a, Rab27b, Rab35), cortical actin regulator cortactin, ADP-ribosylation factor 6 (ARF6), and fusion regulator synaptotagmin-7, and even proteins of the family of endosomal sorting system such as Alix and Tsg101. To deliver their cargo, vesicles must be taken up by the recipient cells. This can occur by interaction between surface receptors and ligands of vesicles and recipient cell, by direct fusion with the plasma membrane, or by endocytosis (Colombo et al., 2014; Maas et al., 2017; Van Niel et al., 2018; Gurunathan et al., 2019; Russell et al., 2019; Lim et al., 2023). Depending on the type of interaction between the recipient cell with the extracellular vesicles, membrane fusion and intracellular fate of sEVs cargo, target molecules in recipient cells can be affected differently. Proteins and nucleic acids can affect the recipient cells either immediately; or after sEVs uptake in case of internal cargoes. Effects of internal cargo on recipient cells are miRNA mediated inhibition or stimulation of translation and transcription mechanisms (Van Niel et al., 2018; Wiklander et al., 2019).

Due to the composition of their membranes, sEVs are relatively stable. Compared to the cell membrane, sEVs membranes are rich in cholesterol, phosphatidylserine, and sphingomyelin, as well and in glycosphingolipids (Figure 2). The lipid bilayer membrane protects the encapsulated cargo from degradation by proteases and nucleases in the extracellular space (Abels and Breakefield, 2016). Vesicles can even cross the blood–brain barrier (Ostrowski et al., 2010).

sEVs can be characterized using electron microscopy or by detection of markers CD9, CD63 and CD81. Current isolation and analytical techniques are unable to efficiently differentiate between sEVs subpopulations, resulting in samples containing various sEVs subtypes. The most widely used isolation method is ultracentrifugation, a simple and cost-effective method based on difference in density and size between particles. Other methods isolate vesicles based on size, shape, or presence of specific surface proteins. These methods include size-exclusion chromatography, ultrafiltration, tangential flow filtration, immunoaffinity capture, and use of microfluidic technologies. sEVs cargo composition can be modified to increase therapeutic potency using electroporation or sonication, and sEVs surface can be modified for cell targeting (Dutta et al., 2021; Lim et al., 2023; Schepici et al., 2023).

### 2.1 Therapeutic potential of sEVs

Since the sEVs contents are influenced by ongoing processes in their cell of origin, both physiological and pathological, many studies have investigated the use of sEVs and their cargo, particularly miRNAs, as potential biomarkers. sEVs could be used to further understand mechanisms involved in progression of various diseases, including cancer metastasis and tumor progression (De Vrij et al., 2015; Urabe et al., 2020), Alzheimer disease (Wang et al., 2017; Sardar Sinha et al., 2018), osteoarthritis and rheumatoid arthritis (Withrow et al., 2016), as well as SCI (Dutta et al., 2021; Yu et al., 2021).

Compared to cell-based therapies, sEVs have several advantages. Lipid bilayer protects encapsulated cargo from degradation and ensures its delivery to the cytosol of recipient cells without triggering an immune response. This makes sEVs a promising tool for delivering specific cargo, such as neuroprotective agents or RNA-based therapeutics, into target cells in a no-invasive manner. Several strategies have been proposed to manipulate sEVs parent cells by molecular engineering techniques to control sEVs loading. Vesicles can be loaded with proteins or RNA molecules either endogenously or exogenously. In the first case, producer cells are used to generate vesicles with desired cargo, usually high concentration of therapeutic agents. In the case of exogenous loading, the cargo is incorporated on or in isolated sEVs. It is also possible to modulate sEVs to express particular surface molecules for modulating biological processes or target specific cell types (György et al., 2014; Tian et al., 2014; Wiklander et al., 2019). Storage of sEVs is possible; however, the exact storage conditions of sEVs require further optimization, since several studies have explored different sEVs storage conditions with inconsistent results. The International Society of Extracellular Vesicles (ISEV) recommends to store isolated sEVs vesicles in −80°C, suspended in phosphate-buffered saline (PBS) in siliconized vessels (Witwer et al., 2013). Addition of trehalose into PBS (PBS-HAT) protects sEVs from cryodamage when stored at-80°C both short-term and long-term (Bosch et al., 2016; Görgens et al., 2022).

Stem cell-derived sEVs present a potential alternative to cellular transplantation, promoting functional recovery and axonal regeneration and possessing anti-inflammatory and immunomodulatory abilities of their parent cells, while having a reduced risk of immunogenicity, uncontrolled differentiation and proliferation when compared to whole cell transplantation.

sEVs derived from various cell types are being investigated for medical and therapeutic applications as a promising alternative to cell transplantation in various diseases, including cancer, diabetes, and neurodegenerative diseases (Zhang et al., 2016; Vogel et al., 2018; Gurunathan et al., 2019; Wiklander et al., 2019). sEVs derived from mesenchymal stem cells (MSC-sEVs) have anti-inflammatory effects, promote angiogenesis, and support of tissue regeneration (Gurunathan et al., 2019). Other stem cell-derived sEVs with similar therapeutic effect as MSC-sEVs include ESCs (Khan et al., 2015), cardiomyocyte progenitor cells (CMPCs) (Vrijsen et al., 2016) and iPSCs (Wang et al., 2015).

However, MSC-sEVs and other non-CNS cell types have been unable to induce key neuroregenerative processes in CNS pathologies, including neurogenesis, neuronal plasticity, and axonal remyelination and regeneration. In comparison to MSC-sEVs, NSC-sEVs have demonstrated better neurotrophic, neuroprotective and anti-inflammatory properties on mouse thromboembolic stroke model (Webb et al., 2018; Mahdavipour et al., 2020), rat TBI model (Elahi et al., 2020; Sun et al., 2020), and SCI (Rong et al., 2019b; Zhong et al., 2020).

### 2.2 miRNA

miRNAs or microRNAs are short, single stranded ncRNA molecules composed of approximately ~22 nucleotides that play a pivotal role in post-transcriptional gene regulation by binding to complementary sequences in the 3-untranslated region (3′ UTR) of target mRNAs, thus suppressing the gene expression, either by inhibiting the protein translation, or by promoting mRNA degradation (Bartel, 2004; Eulalio et al., 2009). A single miRNA can suppress the production of hundreds of proteins by downregulating their mRNA, although its effect is usually quite mild; however, since miRNA can have multiple binding sites within the target mRNA molecule, the overall effect can be enhanced (Lewis et al., 2003; Selbach et al., 2008; Bhalala et al., 2013; O’Brien et al., 2018). miRNAs are powerful regulators in many cellular processes and functions, including development and cell signaling, cellular differentiation and proliferation, metabolism; but they also play a role in progression of pathological conditions of neurological diseases (Bartel, 2004; Branscome et al., 2020). Since miRNAs are released into bodily fluids such as blood, cerebrospinal fluid, urine, and others, detecting alterations in expression of specific miRNAs could be utilized for diagnostic purposes. miRNAs also could serve as targets for therapeutic intervention, or as biomarkers of diseases, such as CNS injuries, neurodegenerative pathologies, and SCI (Bhalala et al., 2013; Rajgor, 2018).

## 3 sEVs and miRNA in CNS

The role of sEVs in the nervous system is very complex. sEVs have been observed to be released from almost all cell types within in CNS, from stem and progenitor cells, neurons, glial cells; and also, from cells of PNS. They participate in neural development, neuroprotection, cellular crosstalk on both local and systemic level, structural remodeling, regeneration of peripheral nerves, and reparative processes after injuries. They are crucial in synaptic neurotransmission and in cell crosstalk within CNS, such as neuron–neuron and glia–neuron communication, regulation of synaptic function, and maintenance of myelination. Furthermore, sEVs and their cargo contribute to neurodegenerative disease and injury progression, including brain tumor, Alzheimer’s disease and other neurodegenerative conditions. They also have functional roles in signaling and regenerative processes occurring during and after SCI. Understanding these processes is still incomplete, however, recent studies have identified cargo within the post-injury circulating sEVs that may be involved in ongoing pathological processes following SCI (Lai and Breakefield, 2012; Kalani and Tyagi, 2015; Krämer-Albers and Hill, 2016; Zappulli et al., 2016; Maas et al., 2017; Holm et al., 2018; Dutta et al., 2021; Khan et al., 2021).

The central nervous system, including brain, spinal cord and peripheral nerves, expresses more miRNAs than any other organ in the body, both in quantity and diversity (Adlakha and Saini, 2014). These miRNAs are crucial in all aspects of CNS processes, from neuronal development and plasticity, cell death, proliferation and differentiation, and pathology-related processes such as inflammation and apoptosis. Moreover, disruption of physiological miRNA regulated processes or of their binding proteins leads to neurodegenerative diseases, and vice versa – several studies have described how miRNA levels are disrupted as a result of CNS injuries (Bhalala et al., 2013; Nieto-Diaz et al., 2014), such as traumatic brain injury (Redell et al., 2010), stroke (Liu et al., 2010), or SCI (Nieto-Diaz et al., 2014; Ning et al., 2014; Zhu et al., 2023); or as a result of neurodegenerative diseases. Many miRNA molecules involved in these pathologies have been identified, for example, miR-9 (F. Chang et al., 2014) and miR-107 (Wang et al., 2008) in Alzheimer’s disease and miR-16-1 in Parkinson’s disease (Zhang and Cheng, 2014). A study on alteration of miRNA expression in sporadic amyotrophic lateral sclerosis (sALS) has shown that a group of miRNAs, namely miR-146a*, miR-524-5p and miRNA-582-3p, were expressed differently in spinal cord when compared to controls. This group of miRNAs interact with and regulate low molecular weight neurofilament (NF-L) mRNA 3′UTR, suggesting their involvement in the selective suppression of NF-L mRNA observed in the spinal motor neurons in amyotrophic lateral sclerosis (ALS) (Campos-Melo et al., 2013).

## 4 sEVs and miRNA in SCI pathology and therapy

### 4.1 miRNA as biomarkers of SCI

miRNAs in the context of SCI have been investigated, both for their role in SCI-related pathological processes, and as potential therapeutic agents or targets for therapeutic intervention. Using next-generation sequencing and SCI animal models, various miRNAs have been identified to play a role in spinal cord and SCI (Liu et al., 2009; Nakanishi et al., 2010; Strickland et al., 2011; Yunta et al., 2012; Bhalala et al., 2013; Hu J. R. et al., 2013; Hu J. Z. et al., 2013; Shi et al., 2017; Ding et al., 2019). Tigchelaar et al. used next-generation sequencing of microRNAs present in serum in porcine SCI model (Tigchelaar et al., 2017), and microRNAs present in cerebrospinal fluid and serum in samples collected from patients suffering from acute traumatic SCI to determine if changes in expression levels of these microRNAs could be utilized as biomarkers of severity of SCI (Tigchelaar et al., 2019). In human patients, alterations of expression levels of several microRNAs in CSF following SCI were identified. In total, Tigchelaar et al. found that 190 microRNAs in the CSF and 19 microRNAs in the serum of human SCI patients were expressed differentially across all time points (24 h, 48 h, 3 days, 4 days, and 5 days post injury) when compared to non-SCI controls. These alterations were described to be dependent on the severity of the injury, with different sets of miRNAs being expressed in patient groups divided based on injury severity according to American Spinal Injury Association (ASIA) Impairment Scale (AIS) classification. Many of these differentially expressed miRNA have been previously identified to be involved in the secondary injury cascade following SCI and investigated as potential biomarkers. These included miR-9, miR-10b, miR-21, and miR-219. Other miRNAs were shown to be strongly associated with injury severity – miR-192-5p, miR-133a-3p, miR-122-5p, miR-194-5p, miR-4792, miR-1246, miR-208b-3p, miR-499a-5p, and miR-148a-3p. In conclusion, severity-dependent pattern of change in microRNA expression in CSF was identified. Moreover, a set of 30 CSF microRNA expressed at 24 h post injury was utilized to determine if grade improvement 6 months post injury could be predicted in AIS A patients with miRNA serving as prognostic markers (Tigchelaar et al., 2019).

Serum exosome markers miR-125b-5p, miR-152-3p and miR-130a-3p have been identified as specific diagnostic markers in acute SCI (Ding et al., 2019), and miR-20a was found to be upregulated in SCI mouse model, targeting proneural gene Neurogenin 1 (Ngn1) (Jee et al., 2012b). miR-21, miR-126, miR-486, miR-20 and miR-133 are involved in crucial SCI processes and have been proposed as potential targets of therapeutic intervention in the SCI treatment (Nieto-Diaz et al., 2014). Jee et al. identified overexpression of miR-486 in motor neurons in SCI lesions and also described how this miRNA induces ROS-mediated neurodegeneration and active inflammatory factor recruitment by suppressing NeuroD6. These findings suggest that miR-486 could be a potential target for therapeutic interventions following SCI (Jee et al., 2012a). Bhalala et al. explored the role of miR-21 in regulation of SCI response, identifying it as a key regulator of astrocytic hypertrophy following SCI. Using SCI mouse contusion model, upregulation in the miR-21 levels in astrocytes in the immediate vicinity of the lesion was observed, whereas miR-21 expression in astrocytes in the uninjured spinal tissue was low. Mildly increased miR-21 levels were detected in the first two weeks following SCI, with a larger increase in expression levels following in later, chronic stage (Bhalala et al., 2012). To further study the role of miR-21 in astrocytes following SCI, transgenic mice were used. These animals were generated to conditionally overexpress either the primary miR-21 transcript in astrocytes, which attenuated the hypertrophic response to SCI; or a miRNA sponge designed to inhibit miR-21 function, which in turn led to hypertrophic phenotype (Bhalala et al., 2012). Hu et al. explored the positive effect of miR-126 in SCI. This miRNA molecule is enriched in endothelial cells and plays a role in vascular integrity. It can also promote angiogenesis during embryonic development and after injury as well. Previously published work of this group (Hu J. Z. et al., 2013) found that miR-126 was downregulated after SCI. In this study, a contusive SCI rat model was used to examine whether upregulation of miR-126 after SCI could have a positive effect on recovery. Results have shown that overexpression of miR-126 promoted angiogenesis, functional recovery after SCI, and inhibited leukocyte extravasation into injured spinal cord. This correlated with downregulation of mRNA and protein expression of miRNA-126 target genes, namely *SPRED1, PIK3R2,* and *VCAM1.* These results suggest an important role of miR-126 in inflammation and angiogenesis following SCI (Hu et al., 2015). Using zebrafish as an animal model for SCI regeneration, the function of miR-133b was investigated. This miRNA molecule targets mRNA of small GTPase RhoA, inhibitor of axonal growth. Following SCI, upregulation of miR-133b was observed in regenerating neurons of the brainstem. Inhibition of miR-133b expression led to a reduction in neuronal axonal regeneration, hindering recovery of locomotor functions; therefore indicating that miR-133b is an important regulator in spinal cord regeneration (Yu et al., 2011).

### 4.2 Stem cells-derived sEVs and miRNAs as therapeutic approaches in the SCI treatment

**Figure 3:**
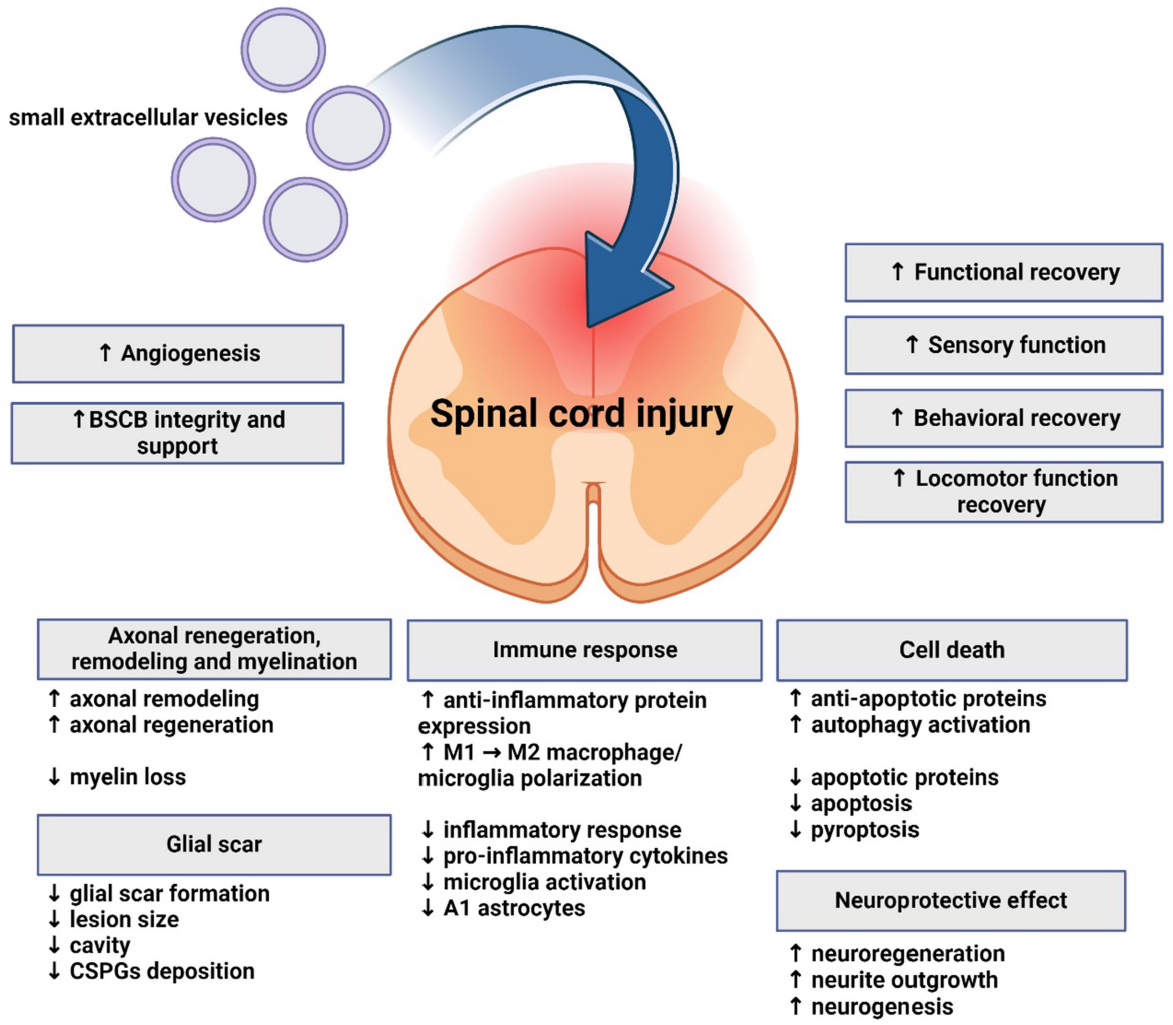
Effects of small extracellular vesicles on spinal cord injury. Small extracellular vesicles support regeneration, functional and motor recovery, angiogenesis, and exhibit neuroprotective and immunomodulatory effects on spinal cord. Axonal regeneration, remodeling and myelination are also influenced. Glial scar formation and lesion size are reduced. Apoptosis is mitigated, while anti-apoptotic and autophagy processes are supported.

As described above, understanding the role sEVs and miRNA in SCI can not only provide new insight into pathological mechanisms of this complex and serious injury, but also help develop new therapeutic strategies. Published studies have evaluated therapeutic potential and mechanism of effect of sEVs derived from various cell types, often modified to express various neuroprotective miRNAs (Figure 3). All studies cited below are included in Supplementary Table S1.

Administration of MSC-derived exosomes promoted angiogenesis and functional recovery in SCI rat model. Cellular apoptosis and inflammation, as well as lesion size were reduced. Following MSC-derived exosome treatment, a significant reduction in the expression levels of pro-apoptotic protein Bcl-2-associated X protein and pro-inflammatory cytokines, while anti-apoptotic and anti-inflammatory proteins were upregulated (Huang et al., 2017). Sung et al. investigated the effect of exosomes isolated from human epidural adipose tissue-derived mesenchymal stem cells (hEpi AD–MSCs) on SCI rat compression model. Exosomes were administered intravenously. Behavioral and histological analysis was performed, as well as miRNA sequencing to elucidate the effects of hEpi AD–MSC-derived exosomes. Results suggest that exosomes mitigated inflammatory response, improved locomotor function, and altered expression of inflammation-related genes in spinal cord tissue post SCI (Sung et al., 2022). Exosomes derived from human placenta MSCs (hPMSCs-Exos) enhanced angiogenesis and improved neurologic function after SCI in a mouse model (Zhang et al., 2020), and intravenously injected MSC-derived exosomes significantly reduced the proportion of neurotoxic A1 astrocytes, presumably through inhibition of nuclear translocation of NF-κB p65, and had neuroprotective effect on injured tissue. Lesion size was decreased as well as expression of pro-inflammatory cytokines (Wang et al., 2018).

Several studies focused on bone MSC-derived exosomes (BMSCs-Exos). These exosomes have demonstrated pro-angiogenic properties, suppressed glial scar formation and inflammation, reduced lesion size, promoted axonal regeneration, and improved functional behavioral recovery after traumatic SCI (Liu et al., 2019). Zhou et al. investigated protective effects of BMSCs-Exos on pericyte pyroptosis in SCI in both *in vitro* and *in vivo.* In *in vitro* experiment, cell culture of pericytes was specifically cultivated to induce SCI conditions. Treatment with BMSCs-Exos led to reduced pericyte pyroptosis and increased pericyte survival rate. *In vivo* experiment used contusive rat model and intravenous administration. As a result, neuronal cell death, BSCB leakage and myelin loss were reduced, myelin arrangement was improved, and locomotor functional recovery was accelerated. Pericyte/endothelial cell coverage on the vascular wall was also increased. Altogether, these results suggest that BMSCs-Exos promoted the survival of pericytes by modulating NOD1-related signaling pathways *in vitro*. BMSCs-Exos improved locomotor recovery following SCI by suppressing pericyte pyroptosis and maintaining BSCB integrity (Zhou Y. et al., 2022). Yu et al. investigated the effects of BMSCs transfected with miRNA-29b, using miR-29b recombinant lentiviral vector along with fluorescent protein reporter gene, and vesicles extracted from these cells when applied intravenously to SCI rat model. When compared to SCI controls, animals treated with miRNA-29b BMSCs and miRNA-29b exosomes both demonstrated improved functional recovery. Reparative processes within the spinal cord were also supported (Yu et al., 2019).

Another study explored transplantation of pericyte-derived exosomes into the SCI mouse model. BSCB is crucial for maintaining integrity and function of nervous system and its disruption in SCI contributes to pathological processes. Pericytes are important for maintenance of blood vessel properties and BSCB integrity, and cooperate closely with capillary endothelial cells, another cell type involved in BSCB formation. Exosomes derived from pericytes can be taken up by endothelial cells easily, and when transplanted to SCI mouse model, these exosomes reduced pathological changes within the spinal cord, improved blood flow and oxygen deficiency after SCI and protected endothelial cells under hypoxic conditions. Hypoxia associated marker HIF-1α levels were significantly reduced. Ability of endothelial cells to regulate blood flow, inhibit apoptosis and protect BSCB was also enhanced. Results also indicate that pericyte-derived exosome could protect against increased BSCB permeability in the early stages of SCI, and related edema formation (Yuan et al., 2019).

Positive effect of sEVs transplantation was also described with vesicles derived from M2 bone marrow-derived macrophages (M2 BMDM-sEVs). Motor function recovery was promoted, and neuronal apoptosis was attenuated. M2 BMDM-sEVs also enhanced autophagy levels of neurons and reduced apoptosis after SCI in a mouse model (Wang J. et al., 2020). Zhou et al. investigated role of miRNAs contained within exosomes derived from M2 microglia in the SCI treatment and its pro-recovery potential in comparison to M0 microglial exosomes. M2-derived exosomes exhibited better promotion of functional recovery behavior and axonal regeneration. Level of pyroptosis, a highly inflammatory type of cell death, of spinal cord neurons after SCI was reduced. miR-672-5p was identified as the most crucial miRNA associated with M2-derived exosomes. M2 exosomes enriched with miR-672-5p could inhibit the AIM2/ASC/caspase-1 signaling pathway by inhibiting AIM2 activity to inhibit neuronal pyroptosis, thus promoting functional behavior recovery in SCI mouse model (Zhou Z. et al., 2022). Ge et al. investigated the effects of M1-polarized bone marrow-derived macrophages (M1-BMDM), a pro-inflammatory macrophage phenotype, on vascular endothelial cells in SCI. Results indicated that infiltrating macrophages after SCI could aggravate the integrity of BSCB by endothelial-to-mesenchymal transition and impairing mitochondrial function in vascular endothelial cells by delivering exosomal miR-155, and subsequently activating the NF-κB pathway by suppressing SOCS6-induced p65 degradation (Ge et al., 2021).

Not only can sEVs derived from M1 and M2 macrophages affect SCI processes, but phenotype of macrophages present in the injured spinal cord can be polarized by transplanted sEVs. Intravenous injection of MSC-sEVs derived from human umbilical cord (hucMSCs) into SCI mouse model demonstrated effect on macrophage polarization from pro-inflammatory M1 phenotype to alternatively activated, pro-repair and anti-inflammatory M2 phenotype; functional recovery was promoted as well, and inflammation at the site of injury was reduced (Sun et al., 2018).

Transplantation of MSC-sEVs requires further optimalization, as the cell culture conditions often do not correspond to the conditions that prevail in the damaged spinal cord tissue. In most studies, MSCs are cultivated under normoxia *in vitro*, however, this differs greatly from hypoxic microenvironment of SCI. Liu et al. investigated whether exosomes derived from MSCs under hypoxia (HExos) have greater effects on functional behavioral recovery than those under normoxia (Exos) following SCI in a mouse model. HExos enriched in miR-216a-5p promoted functional behavioral recovery by shifting microglial polarization from M1 to M2 phenotype *in vivo* and *in vitro*. TLR4 was identified as the target downstream gene of miR-216a-5p. Results indicated that TLR4/NF-κB/PI3K/Akt signaling pathway may be involved in the modulation of microglial polarization by hypoxic exosomal miR-216a-5p (Liu et al., 2020).

Lankford et al. investigated trafficking of MSC-sEVs into the site of the injury, and cell types targeted by their effect. Intravenously administered, fluorescently labeled MSC-derived exosomes were detected within the injured spinal cord, and not in the uninjured tissue. M2 macrophages at the SCI site were identified as specific targets of MSC-derived exosomes. Labeled exosomes were taken up by macrophages within the SCI lesion and possibly by the cells in the spleen of experimental animals, which was notably reduced in weight in the SCI rat, compared to control animals. This weight reduction suggests an increased mobilization of monocytes following SCI (Lankford et al., 2018). Previous study from this group demonstrated that intravenous injection of MSCs has indeed positive effect after contusive SCI, improving functional recovery; however, MSCs were not detected in injured spinal cord, but in lungs. This supports the idea that the therapeutic effects of MSCs are not mediated by the transplanted cells themselves, but by sEVs released from the cells (Matsushita et al., 2015).

The most used method of sEVs administration is tail vein injection. Other methods of administration that are less invasive and more effective are being explored. A pre-clinical SCI study using intranasal administration reported beneficial effect of MSC-derived vesicles loaded with phosphatase and tensin homolog small interfering RNA (PTEN-siRNA; ExoPTEN). A member of mTOR signaling pathway, PTEN is expressed in neurons and regenerating axons and is crucial for control the regeneration of corticospinal neurons. MSC-sEVs loaded with ExoPTEN were administered intranasally to rats with complete SCI. These MSC-sEVs were capable of crossing blood brain barrier, moving into the spinal cord, and targeting the site of the SCI. Migration of MSC-sEVs was attracted by neuroinflammation, with high affinity to neurons at the site the injury. Intranasal ExoPTEN attenuated expression of PTEN at the site of the injury. Enhanced regeneration of corticospinal axons and angiogenesis, while reducing gliosis and neuroinflammation. Treated animals have shown improved motor and sensory functions. Potentially, this method of therapeutic approach could improve structural and electrophysiological conduction, as well as functional recovery in rats with complete SCI. Other advantages of this approach included its non-invasive administration and lesion specific targeting (Guo et al., 2019).

Delivery of miRNA to the site of the injury is limited and requires optimization, since miRNA delivery *in vivo* has very low efficiency. Culture cells can be modified using techniques of gene engineering to produce vesicles enriched with miRNA molecules with neuroprotective properties or other functional effects and serve as delivery agents. Wang et al. investigated the positive effect of hucMSC-derived exosomes transfected with miR-199a-3p/145-5p in SCI rat model. TrkA ubiquitination was affected and the NGF/TrkA signaling pathway was promoted (Wang et al., 2021). Ren et al. utilized SCI rat model to investigate the effect of exosomes derived from miR-133b-modified adipose-derived stem cells (ADSCs) on neurological function after SCI and its mechanism. Results demonstrated that the modified adipose MSC-derives exosomes promoted recovery after SCI (Ren et al., 2019).

Huang et al. used MSC-derived exosomes as delivery vehicles for miR-126 into the injured spinal cord, thus improving functional recovery. Authors suggested that angiogenesis was promoted through miR-126 suppression of its target genes *SPRED1* and *PIK3R2*. miR-126 also supported neurogenesis, suppressed inflammation after SCI, and reduced apoptosis and lesion size (Huang et al., 2020). Kang et al. investigated how miR-21 derived from MSC exosomes regulates neuronal differentiation and death in patients suffering from SCI, demonstrating that miR-21 inhibited neuronal apoptosis by regulating expression of PTEN and PDCD4, molecules with pro-apoptotic effects and targets of miR-21. Exosomes collected from MSC supernatant transfected with miR-21 or PTEN siRNA promoted recovery after SCI in a rat model (Kang et al., 2019). Xu et al. achieved similar results using human MSC-derived sEVs and differentiated PC12 cells-derived sEVs (Xu et al., 2019).

Phosphatase and tensin homolog pseudogene 1 (PTENP1) can suppress apoptosis of neurons. A study investigated its role in SCI recovery, using exosomes derived from MSCs transfected with PTENP1 short hairpin RNA (shRNA) as a type of novel biomarkers in the treatment of SCI. Results demonstrated that exosomes suppressed apoptosis of neurons, and that PTENP1 was involved in the recovery of SCI by regulating the expression of miR-19b and miR-21, both of which negatively regulate expression of PTEN (Wang Z. et al., 2020). Chen et al. investigated therapeutic effects of miR-26a-modified MSC-derived exosomes (Exos-26a) following SCI, with PTEN being miR-26a target in regulation of angiogenesis, tumorigenesis and other processes. miR-26a-overexpressing exosomes demonstrated potential to promote axonal regeneration and attenuate glial scarring following SCI by inhibiting PTEN and subsequent activation of Akt/mTOR signaling cascades (Chen et al., 2021).

NSC-sEVs have demonstrated promising outcomes in the treatment of central nervous system (CNS) pathologies, primarily attributed to their capacity to regulate autophagy. Rong et al. were the first ones to investigate the potential therapeutic effect of NSC-sEVs in SCI. NSC-sEVs derived from culture medium of mouse NSCs have demonstrated potential to significantly mitigate the extent of SCI, reducing neuronal apoptosis and microglia activation. Furthermore, administration of NSC-sEVs inhibited neuroinflammation, reduced lesion size, and promoted functional recovery in SCI model rat by activating autophagy. This was mediated by increasing the expression of the autophagy marker proteins LC3B and beclin-1, thus promoting autophagosome formation. Expression of pro-apoptotic proteins and pro-inflammatory cytokines was also significantly reduced (Rong et al., 2019b). Similar results were observed by Zhong et al., who explored the influence of exosomes derived from NSCs (NSCs-Exos) on the spinal cord microvascular regeneration after SCI, particularly on spinal cord microvascular endothelial cells (SCMECs). This cells’ limited plasticity is one of the major causes for bad prognosis following SCI. Result have shown that NSCs-Exos promoted angiogenic activities of SCMECs and recovery of neurological function following SCI. Moreover, spinal cord cavity was reduced, and Basso mouse scale scores in SCI mice were improved. This positive effect was mediated through transfer of exosomal VEGF-A (Zhong et al., 2020). Following their previous study (Rong et al., 2019b), Rong et al. further investigated mechanisms by which NSC-sEVs repair spinal cord injury through modulation of autophagy. They found that NSC-sEVs contain 14–3-3t protein, member of 14–3-3 protein family. These highly conserved proteins are involved in many cellular processes. Different subtypes have regulatory functions on autophagy in neurodegenerative diseases. It was revealed that the overexpression of 14–3-3t in NSC-sEVs promoted autophagy, which in turn enhanced the anti-apoptotic and anti-inflammatory effects of NSC-sEVs *in vitro* and *in vivo*. Knockdown of 14–3-3t in NSC-sEVs attenuated the anti-apoptotic and anti-inflammatory effects of NSC-sEVs *in vitro*. Results also suggested that 14–3-3t derived from NSC-sEVs activates autophagy by interacting with protein Beclin-1, which sheds light on the mechanism of effect of NSC-sEVs in SCI (Rong et al., 2019a).

Similar to MSCs, NSCs and NSC-sEVs can be modified to enhance their therapeutic and regenerative properties. Using SCI mouse model, Jiang et al. observed that exosomes derived from differentiated neurons and enriched with miR-124-3p promoted functional recovery through suppression of activation of pro-inflammatory A1 astrocytes and M1 microglia, through regulation of myosin heavy chain 9 (MYH9) in particular (Jiang et al., 2020). Ma et al. examined NSC-derived exosomes and miRNAs isolated from them in SCI therapy. When cultivated in the presence of insulin growth factor-1 (IGF-1), which promotes neural proliferation and regeneration, NSC-derived exosomes expressed enhanced neuroprotective effects in SCI. Apoptosis was inhibited, neuroinflammation was suppressed and neuroregeneration was promoted in an miR-219a-2-3p-dependent manner (Ma et al., 2019).

In addition to NSC-sEVs, vesicles derived from other cell types of neuronal origin have been examined for their therapeutic potential in SCI. Mohammed et al. investigated the effect of intrathecal transplantation of SVZ-derived sEVs on the NOD-like receptor protein-3 (NLRP3) inflammasome complex formation in SCI rats. sEVs promoted functional and motor recovery, reduced apoptosis and regenerated neural cells following SCI through suppression of NLRP3 inflammasome complex formation (Mohammed et al., 2020). Another study utilized Schwann cell-derived exosomes (SCDEs) that were injected into mice with induced SCI. SCDEs promoted functional recovery by decreasing CSPG deposition via an increase in TLR2 expression on astrocytes through the NF-κB/PI3K signaling pathway (Pan et al., 2021).

## 5 Conclusion

Despite new understanding of pathological mechanisms of SCI and tremendous advancement in therapeutic strategies in last years, there is still no effective treatment available. Promising therapeutic approaches include stem cell transplantation, which has a well-documented supportive and immunomodulating effect, presumably mediated by sEVs secreted by cells, and by miRNAs contained within them.

Studies have shown that application of sEVs derived from various stem cell sources and enriched in protective miRNAs have a positive effect on tissue regeneration following SCI; reducing neuroinflammation, astrogliosis and lesion size while improving motor and functional recovery by attenuating neuronal apoptosis, and supporting neurogenesis, angiogenesis and axonal remodeling. Furthermore, vesicles derived from CNS cell types have demonstrated regenerative and neuroprotective properties of their parent cells, improving recovery of locomotor functions in SCI animal models, and are considered more suitable for the treatment of CNS pathologies than MSC-sEVs.

However, there are still many unknowns and technical challenges, the main one being the production sEVs in sufficient quantity and purity for therapeutic applications. Isolated sEVs from biological fluids are very heterogenous and impure and include many different subpopulations of sEVs. Standardization and optimization of production, isolation and analysis is required to improve the quality of isolated particles. The exact mechanisms by which sEVs influence target cells, as well as the effects of their encapsulated miRNAs, have not yet been fully elucidated. More studies based on relevant animal models focusing on cell-derived sEVs transplantation are necessary to properly elucidate mechanisms of their therapeutic effect.

In conclusion, it is safe to say that despite technical limitations and need for further research into the possibilities of therapeutic application, stem cell-derived sEVs and miRNA-based therapeutic approaches hold a great promise in the SCI treatment.

## Author contributions

KS: Funding acquisition, Writing – original draft, Writing – review & editing, Data curation, Visualization. NR: Conceptualization, Funding acquisition, Supervision, Writing – review & editing.
