# Supplementary material for: The role of small extracellular vesicles and microRNA as their cargo in the spinal cord injury pathophysiology and therapy
Source: Front Neurosci. 2024 May 7;18:1400413. doi: 10.3389/fnins.2024.1400413 (PMC11106386; doi:10.3389/fnins.2024.1400413)
Supplement: Supplementary file 1 [file Table_1.docx]

Supplementary Material

**Table 1. Therapeutic effects of different cell types and their vesicles derived from various sources in SCI animal models.**

mesenchymal stem cells (MSCs); human epidural adipose tissue MSCs (hEpi AD–MSCs); human placenta-derived MSCs (hPMSCs); bone marrow MSCs (BMSCs); spinal cord microvascular endothelial cells (SCMECs); bone marrow-derived macrophages (M2 BMDM); human umbilical cord MSCs (hucMSCs); green fluorescent protein MSCs (GFP-MSCs); adipose-derived stem cells (ADSCs); bone marrow-derived macrophages (BMDMs)

| Cell source | Animal Model | Method of administration | Effects | Ref |
| --- | --- | --- | --- | --- |
| Primary culture of BMSCs (rat) | Rat, contusion model | Tail vein injection | Promoted functional recovery and angiogenesis; reduced expression of apoptotic proteins and pro-inflammatory cytokines; upregulated anti-apoptotic and anti-inflammatory proteins | (Huang et al., 2017) |
| hEpi AD-MSCs | Rat, compression model | Tail veil injection | Improved locomotor function; suppressed inflammatory response | (Sung et al., 2022) |
| hPMSCs | Mouse, contusion model | Direct injection into the SCI epicenter | Improved locomotor and sensory function; enhanced angiogenesis in endothelial cells | (Zhang et al., 2020) |
| Primary culture of MSCs (rat) | Rat, contusion model | Tail vein injection | Anti-inflammatory and neuroprotective effects; decreased A1 astrocytes; decreased lesion size | ( Wang et al., 2018) |
| Primary culture of BMSCs (rat) | Rat, contusion model | Tail vein injection | Improved functional and behavioral recovery; reduced lesion size and glial scar formation; promoted axonal regeneration and angiogenesis | ( Liu et al., 2019) |
| Primary culture of BMSCs (rat) | Rat, contusion model | Tail vein injection | Improved locomotor recovery and BSCB integrity; suppressed pericyte pyroptosis; reduced myelin loss | (Zhou Y. et al., 2022). |
| miRNA-29b-modified BMSCs | Rat, contusion model | Intravenous, tail vein injection | Improved functional recovery and reparative processes | (Yu et al., 2019) |
| Pericytes; primary culture of SCMECs (mouse) | Mouse, contusion model | Tail vein injection | Improved functional and behavioral recovery, protection of the BSCB and endothelial cells; mitigated apoptotic response | (Yuan et al., 2019) |
| Primary culture of M2 BMDMs (mouse) | Mouse, contusion model | Tail vein injection | Improved functional recovery; reduced neuronal apoptosis | (Wang J. et  al., 2020) |
| M2 microglia | Mouse | - | Improved functional recovery; reduced level of pyroptosis in spinal cord neurons | (Zhou Z. et al., 2022) |
| Primary culture of BMDMs (mouse) | Mouse, contusion model | Tail vein injection | Infiltrated macrophages aggravated BSCB integrity breakdown after SCI | (Ge et al., 2021) |
| hucMSCs | Mouse, contusion model | Tail vein injection | Promoted functional recovery and anti-inflammatory macrophage polarization | (Sun et al., 2018) |
| BMSCs | Mouse, contusion model | Tail vein injection | Promoted functional behavioral recovery; microglia polarization shifted from M1 to M2; regulated neuroinflammatory response and microglia activation | (Liu et al., 2020) |
| Primary culture of MSCs (rat) | Rat, contusion model | Intravenous injection | Association with M2 macrophages; administered exosomes present only in injured spinal cord | (Lankford et al., 2018) |
| Primary culture of bone marrow derived GFP-MSCs (rat) | Rat, contusion model | Microinjections into the injured area | Improved functional recovery, locomotor function and stabilization of the BSCB | (Matsushita et al., 2015) |
| Human BMSCs | Rat, transection model | Intranasal administration | Enhanced functional and locomotor recovery and regeneration; reduced gliosis and neuroinflammation | (Guo et al., 2019) |
| hucMSCs transferred with miR-199a-3p/145-5p | Rat | Tail vein injection | Promoted locomotor and functional recovery, angiogenesis, neurogenesis, axonal remodeling, and neurite outgrowth; decreased inflammation at the lesion site | (Wang et al., 2021) |
| miR-133b-modified ADSCs | Rat, compression model | - | Improved recovery of neurological function through affecting the signaling pathway related to axon regeneration | (Ren et al., 2019) |
| Primary culture of miR-126-modified MSCs (rat) | Rat, contusion model | Tail vein injection | Improved functional recovery; supported angiogenesis and neurogenesis; reduced apoptosis, lesion size, and inflammation | (Huang et al., 2020) |
| MSCs transfected with miR-21 or PTEN siRNA | Rat, contusion model | Intravenous injection | Promoted recovery; inhibited neuronal apoptosis | (Kang et al., 2019) |
| MSCs; differentiated PC12 cells, | Rat, contusion model | Intravenous injection | Inhibited the expression of PTEN; regulated apoptosis and differentiation of neurons | (Xu et al., 2019) |
| Primary culture of miRNA-29b-modified BMSCs | Rat, contusion model | Intravenous injection | Improved motor function, SCI repair and outcome; regulated proteins involved in neuronal regeneration | (Wang Z. et al., 2020) |
| Primary culture of MSCs (rat); PC12 cells | Rat, contusion model | Intravenous injection | Promoted axonal regeneration and neurogenesis; attenuated glia scarring | (Chen et al., 2021) |
| Primary culture of neural stem cells (fetal mouse) | Rat, contusion model | Tail vein injection | Improved motor function and functional recovery; reduced lesion size and SCI extent, inflammation, neuronal apoptosis and microglia activation; supported autophagy activation and neuroprotection | (Rong et al., 2019b) |
| Primary culture of SCMECs (mouse) | Mouse, contusion model | Tail vein injection | Improved motor function; reduced lesion size and cavity; promoted angiogenesis and recovery of neurological function | (Zhong et al., 2020) |
| Primary culture of neural stem cells (fetal mouse) | Rat, contusion model | Tail vein injection | Improved motor function; reduced lesion size and inflammation; promoted autophagy and neuroprotection, anti-apoptotic and anti-inflammatory effects | (Rong et al., 2019a) |
| Primary neuronal cultures | Mouse, contusion model | Tail vein injection | Improved motor function, functional and behavioral recovery; reduced lesion size and activation of pro-inflammatory M1 and A1 | (Jiang et al., 2020) |
|  |  |  |  |  |
| Primary culture of neural stem cells (fetal rat) | Rat, compression model | Tail vein injection | Improved motor function, neuroprotection and neuroregeneration; reduced apoptosis and neuroinflammation | (Ma et al., 2019) |
| Primary culture of neural stem cells (rat) | Rat, compression model | Intrathecal injection | Promoted functional and motor recovery; supported neuroprotection; reduced lesion size and apoptosis; regenerated neural cells following SCI by suppressing inflammasome complex formation | (Mohammed et al., 2020) |
|  |  |  |  |  |
| Primary culture of Schwann cells (mouse) | Mouse, contusion model | Tail vein injection | Promoted functional recovery; decreased CSPGs deposition | (Pan et al., 2021) |
